# RETRACTION: Purpurogallin, a Natural Phenol, Attenuates High‐Mobility Group Box 1 in Subarachnoid Hemorrhage Induced Vasospasm in a Rat Model.

**DOI:** 10.1155/ijvm/9767252

**Published:** 2026-04-01

**Authors:** International Journal of Vascular Medicine

**RETRACTION**: C.‐Z. Chang, C.‐L. Lin, S.‐C. Wu, and A.‐L. Kwan, “Purpurogallin, a Natural Phenol, Attenuates High‐Mobility Group Box 1 in Subarachnoid Hemorrhage Induced Vasospasm in a Rat Model,” *International Journal of Vascular Medicine* 2014, 254270. https://doi.org/10.1155/2014/254270.

The above article, published online on 17 November 2014 in Wiley Online Library (wileyonlinelibrary.com), has been retracted by agreement between the journal’s Editor Sreeja Dattachoudhury; and John Wiley & Sons Ltd. A third party reported that images in Figure 1 in this article had been published in other articles by some of the authors. Each image was reported differently in the various publications. Additionally, an investigation by the publisher found that the beta‐actin bands in Figure 3 had been published in another article by some of the same authors [Chang et al. 2015 (10.1186/s12950-015-0074-3)] and that the GAPDH band in Figure 4 had been published in a separate article, also by some of the same authors [Chang et al. 2015 (https://link.springer.com/article/10.1186/s12993-015-0074-8)].

The authors did not respond to an inquiry and request for original data by the publisher.

The retraction has been agreed to because the evidence of image duplication fundamentally compromises the editors’ confidence in the results presented. The authors did not respond to our notice regarding the retraction.

